# Chromogranin A plasma levels predict mortality in COVID-19

**DOI:** 10.1371/journal.pone.0267235

**Published:** 2022-04-25

**Authors:** Rebecca De Lorenzo, Clara Sciorati, Giuseppe A. Ramirez, Barbara Colombo, Nicola I. Lorè, Annalisa Capobianco, Cristina Tresoldi, Bio Angels for COVID-BioB Study Group, Daniela M. Cirillo, Fabio Ciceri, Angelo Corti, Patrizia Rovere-Querini, Angelo A. Manfredi

**Affiliations:** 1 Division of Immunology, Transplantation & Infectious Diseases, IRCCS San Raffaele Scientific Institute, Milan, Italy; 2 Vita-Salute San Raffaele University, Milan, Italy; 3 Tumor Biology & Vascular Targeting Unit, Division of Experimental Oncology, IRCCS San Raffaele Scientific Institute, Milan, Italy; 4 Emerging Bacterial Pathogens Unit, IRCCS San Raffaele Scientific Institute, Milan, Italy; 5 Hematology & Bone Marrow Transplant, IRCCS San Raffaele Scientific Institute, Milan, Italy; Kaohsuing Medical University Hospital, TAIWAN

## Abstract

**Background:**

Chromogranin A (CgA) and its fragment vasostatin I (VS-I) are secreted in the blood by endocrine/neuroendocrine cells and regulate stress responses. Their involvement in Coronavirus 2019 disease (COVID-19) has not been investigated.

**Methods:**

CgA and VS-I plasma concentrations were measured at hospital admission from March to May 2020 in 190 patients. 40 age- and sex-matched healthy volunteers served as controls. CgA and VS-I levels relationship with demographics, comorbidities and disease severity was assessed through Mann Whitney U test or Spearman correlation test. Cox regression analysis and Kaplan Meier survival curves were performed to investigate the impact of the CgA and VS-I levels on in-hospital mortality.

**Results:**

Median CgA and VS-I levels were higher in patients than in healthy controls (CgA: 0.558 nM [interquartile range, IQR 0.358–1.046] vs 0.368 nM [IQR 0.288–0.490] respectively, p = 0.0017; VS-I: 0.357 nM [IQR 0.196–0.465] vs 0.144 nM [0.144–0.156] respectively, p<0.0001). Concentration of CgA, but not of VS-I, significantly increased in patients who died (n = 47) than in survivors (n = 143) (median 0.948 nM [IQR 0.514–1.754] vs 0.507 nM [IQR 0.343–0.785], p = 0.00026). Levels of CgA were independent predictors of in-hospital mortality (hazard ratio 1.28 [95% confidence interval 1.077–1.522], p = 0.005) when adjusted for age, number of comorbidities, respiratory insufficiency degree, C-reactive protein levels and time from symptom onset to sampling. Kaplan Meier curves revealed a significantly increased mortality rate in patients with CgA levels above 0.558 nM (median value, log rank test, p = 0.001).

**Conclusion:**

Plasma CgA levels increase in COVID-19 patients and represent an early independent predictor of mortality.

## Introduction

Chromogranin A (CgA) is a 439-residue-long protein member of the secretogranin family [1]. It is expressed and secreted by various normal and neoplastic endocrine/neuroendocrine cells and expressed by myocardial cells and immune cells [2–4]. Within the cells, CgA has a role in the regulation of calcium homeostasis and granule biogenesis [5]. Intracellular and secreted CgA is cleaved by proteases, including prohormone convertases, furin, cathepsin, plasmin and thrombin, to generate biologically active fragments [6] such as Vasostatin I (VS-I) [7], a 76-residue long polypeptide that regulates vascular homeostasis and heart function [8].

High plasma levels of CgA have been first described in patients with neuroendocrine tumors [9], but have been also found in patients affected by heart failure, arterial hypertension (HTN), renal failure, rheumatoid arthritis, giant cell arteritis, diabetes mellitus (DM), inflammatory bowel diseases and sepsis [10, 11]. Elevated CgA plasma levels are associated with mortality risk in patients with myocardial infarction, acute coronary syndrome and heart failure [12]. High levels of CgA and VS-1 have been also described in fatal cases of systemic inflammatory response syndrome [13, 14]. The secreted CgA fragments contributes to the host defense and are part of the acute phase response [15], yielding upon proteolytic processing moieties that have anti-microbial proprieties [16], activate neutrophils [17] regulate macrophage polarization [18] and monocyte chemotaxis [19].

Coronavirus disease 2019 (COVID-19) presents with various degrees of severity. Most patients are asymptomatic or experience symptoms that reflect upper or lower airway involvement. In other patients, a less effective innate immune response fails to limit viral replication, eventually resulting in acute respiratory distress syndrome, metabolic derangements and multiorgan failure [20–22]. Age and pre-existing comorbidities including cardiovascular and respiratory diseases, diabetes mellitus (DM) and hypertension (HTN) are risk factors for severe COVID-19 [23]. In turn, COVID-19 cardiovascular, neurological, renal, and vascular complications are associated with mortality [24, 25].

The aims of this study were to assess whether the release of CgA and VS-I in circulation is part of the early host response in COVID-19 and whether these molecules, measured at disease onset, might predict adverse outcomes.

## Methods

### Patients and study design

This retrospective and prospective study included one hundred ninety patients. The inclusion criteria were: age ≥18 years, confirmed SARS-CoV-2 infection, clinical and/or radiological signs of COVID-19 pneumonia, admission to the Emergency Department of San Raffaele University Hospital, Milan, from March 18 to May 5, 2020, blood sampling at hospital admission. No exclusion criteria were applied [26]. COVID-19 was diagnosed based on a positive real-time reverse-transcriptase polymerase chain reaction (RT-PCR) from a nasopharyngeal swab in the presence of clinical and/or radiologic findings of COVID-19 pneumonia. Blood samples were collected at hospital admission and stored in a dedicated institutional biobank [27]. Detailed demographic, laboratory and clinical data from all patients were recorded in a specific electronic case record form. Patients were prospectively followed until hospital discharge or death. All patients signed an informed consent. The study is compliant with the declaration of Helsinki, was approved by the Hospital Ethics Committee (protocol no. 34/int/2020) and registered on ClinicalTrials.gov (NCT04318366). Forty age- and sex-matched volunteers served as healthy controls (HC).

### CgA and VS-I measurement

Plasma-EDTA samples were obtained from venous blood by double centrifugation, according with the Institutional Biobank procedures [27]. The samples were then immediately transferred at –80 °C and 24–72 hours later stored in liquid nitrogen until usage. Plasma were transferred to research laboratory the day of the analysis, thawed and inactivated using tri-(n-butyl) phosphate and Triton X-100 (Sigma) (0.3% and 1% respectively) for 2 hours [28, 29]. CgA and VS-I were measured by enzyme-linked immunosorbent assay as previously described [30]. Samples were diluted 1:10 for CgA assay and or 1:5 for VS-I assay.

### Variables and outcome

The following variables were included: age, sex, selected pre-existing comorbidities (HTN, coronary artery disease [CAD], DM, chronic obstructive pulmonary disease [COPD], chronic kidney disease [CKD], active neoplasia), clinical and laboratory data at hospital admission (the ratio of arterial oxygen partial pressure [PaO_2_] in mmHg to fractional inspired oxygen [FiO_2_] expressed as a fraction [PaO_2_/FiO_2_], neutrophil to lymphocyte ratio [NLR], concentration of C-reactive protein [CRP] and of lactate dehydrogenase [LDH]). Time from symptom onset to blood sampling, rate of hospitalization, length of stay, therapy administered during hospital stay, transfer to the intensive care unit (ICU) and death were recorded. In-hospital mortality was used as primary outcome.

### Statistical analysis

Absolute counts (percentage) and median (interquartile range [IQR]) were used to express categorical and continuous variables, respectively. The only exception was the number of comorbidities that was expressed as mean (standard deviation). Differences in categorical and continuous variables between groups were assessed using Chi-squared or Fisher test, as appropriate and Mann-Whitney U test, respectively. Spearman’s correlation test was used to investigate the relationships between continuous variables. Multivariable Cox regression analysis was performed to investigate the impact of CgA on the primary outcome when adjusting for confounders. Variables that showed substantial redundancy with other variables (i.e. CRP vs. NLR) were excluded from the multivariable regression analysis to prevent model overfitting. Similarly, chronic proton pump inhibitor (PPI) therapy, which is associated with high levels of CgA in plasma [31], was not included in the multivariable model. Statistical analyses were performed using R statistical package (version 4.0.0. R Foundation for Statistical Computing, Vienna, Austria), with a two-sided significance level set at p <0.05.

## Results

### Patient characteristics

Demographic, clinical and laboratory characteristics of the patients (n = 190) are summarized in Table 1. Blood samples were obtained at hospital admission (median [IQR] time from admission to blood draw was 1 [0–1] days). 185 patients (97.3%) had not received any COVID-19-related treatment prior to sample collection. 32 patients (17%) were on chronic PPI therapy. Most patients were males (64%) and median (IQR) age was 61.5 (49.9–72.1) years. More than half of patients had pre-existing comorbidities (52%), the most frequent being HTN (41%). 152 patients (80%) were hospitalized for a median (IQR) time of 15 (9–29) days. 41 (21%) patients were transferred to the intensive care unit (ICU) and 47 (25%) died. Survival time in patients who died was 12 (5–21) days. As expected, patients who died were older and with a higher burden of comorbidities. In addition, patients with a fatal outcome had lower levels of PaO_2_/FiO_2_ and higher levels of CRP, NLR and LDH at hospital admission (all p<0.001, Table 1). Steroids and LMWH were, as expected, administered more frequently during hospital stay in non-survivors due to a more severe disease burden (both p<0.05).

**Table 1:** General and disease characteristics of COVID-19 patients.

	Overall (n = 190)	Dead (n = 47)	Alive (n = 143)	P value
Age (years)	61.5 (49.9–72.1)	72.6 (62.6–79.6)	57.7 (48.5–67.2)	<0.0001
Male sex	122 (64)	30 (63)	92 (64)	>0.99
Comorbidities				
≥1 comorbidity	98 (52)	35 (74)	63 (44)	0.00056
Number of comorbidities	0.9 (0.008)	1.5 (0.18)	0.7 (0.082)	<0.0001
HTN	78 (41)	27 (57)	51 (36)	0.014
COPD	10 (5)	6 (13)	4 (3)	0.023
CAD	22 (11)	12 (25)	10 (7)	0.0015
DM	39 (20)	15 (32)	24 (17)	0.043
CKD	17 (9)	9 (19)	8 (6)	0.011
Active neoplasia	6 (3)	3 (6)	3 (2)	0.32
Time from symptom onset to sampling (days)	8 (4–11)	5 (2–8)	8 (5–11)	<0.0001
*At hospital admission*				
PaO_2_/FiO_2_	278.5 (190.5–334.6)	159.1 (79.2–266.6)	304.5 (238.1–348.0)	<0.0001
NLR	5.3 (3.5–8.5)	9.9 (5.4–13.5)	4.8 (3.2–7.2)	<0.0001
CRP (mg/dL)	78.8 (30.3–153.3)	151.1 (79.4–213.6)	68.7 (20.4–125.6)	<0.0001
LDH (U/L)	383 (275–493.5)	440 (365–627)	354 (271.5–466.5)	0.00040
Hospitalization	152 (80)	46 (98)	106 (74)	<0.0001
Length of stay (days)†	12 (4–22)	12 (3–24)	12 (3–24)	0.44
ICU transfer	41 (22)	22 (47)	19 (13)	<0.0001
Death	47 (25)	47 (100)	0	-
*Therapy during hospitalization*				
Steroids	43 (23)	22 (47)	21 (15)	<0.0001
LMWH	86 (45)	25 (53)	61 (43)	<0.020

Categorical variables were expressed as count (percentage), while continuous variables as median (interquartile range), with the exception of the number of comorbidities which was expressed as mean (standard deviation).

^†^ Calculated as the time from hospital admission to death or discharge.

Abbreviations: PaO_2_/FiO_2_, ratio of arterial oxygen partial pressure to fractional inspired oxygen; NLR, neutrophil to lymphocyte ratio; ICU, intensive care unit, LMWH, low molecular weight heparin

### CgA and VS-I plasma levels in COVID-19

**Fig 1:**
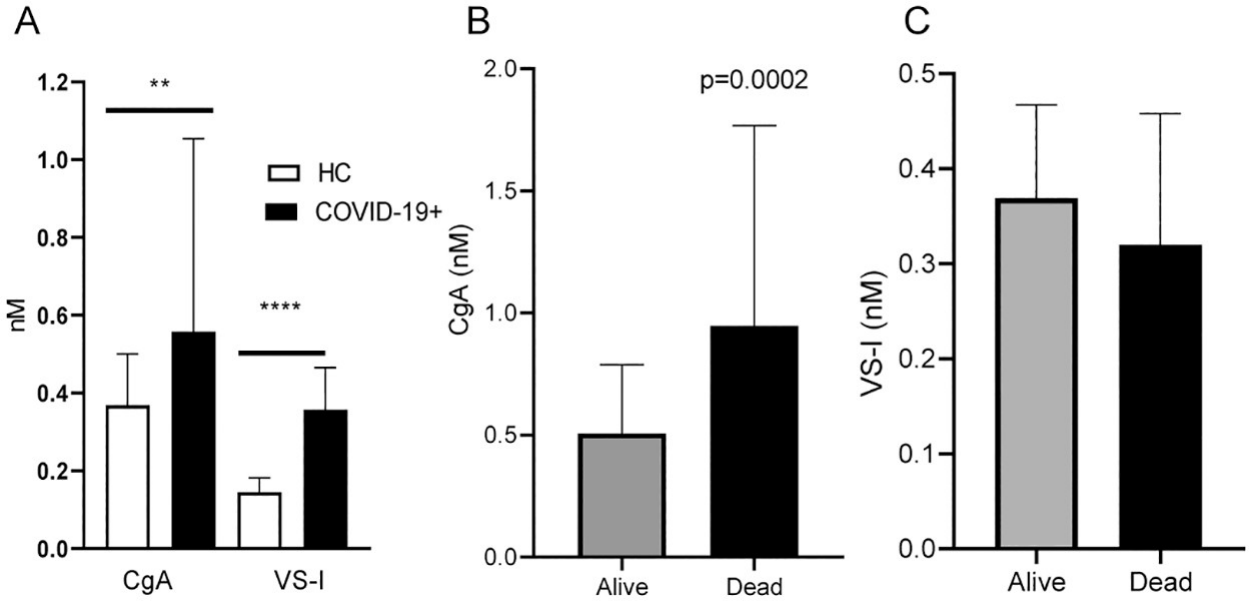
Panel A: CgA and VS-I plasma levels in age- and sex-matched healthy controls (HC) and COVID-19 patients at hospital admission. Panel B: CgA plasma levels in COVID-19 patients with favorable outcome (Alive) or who died (Dead). Panel C: VS-I in Alive or Dead patients. ** p<0.001.

**Fig 2:**
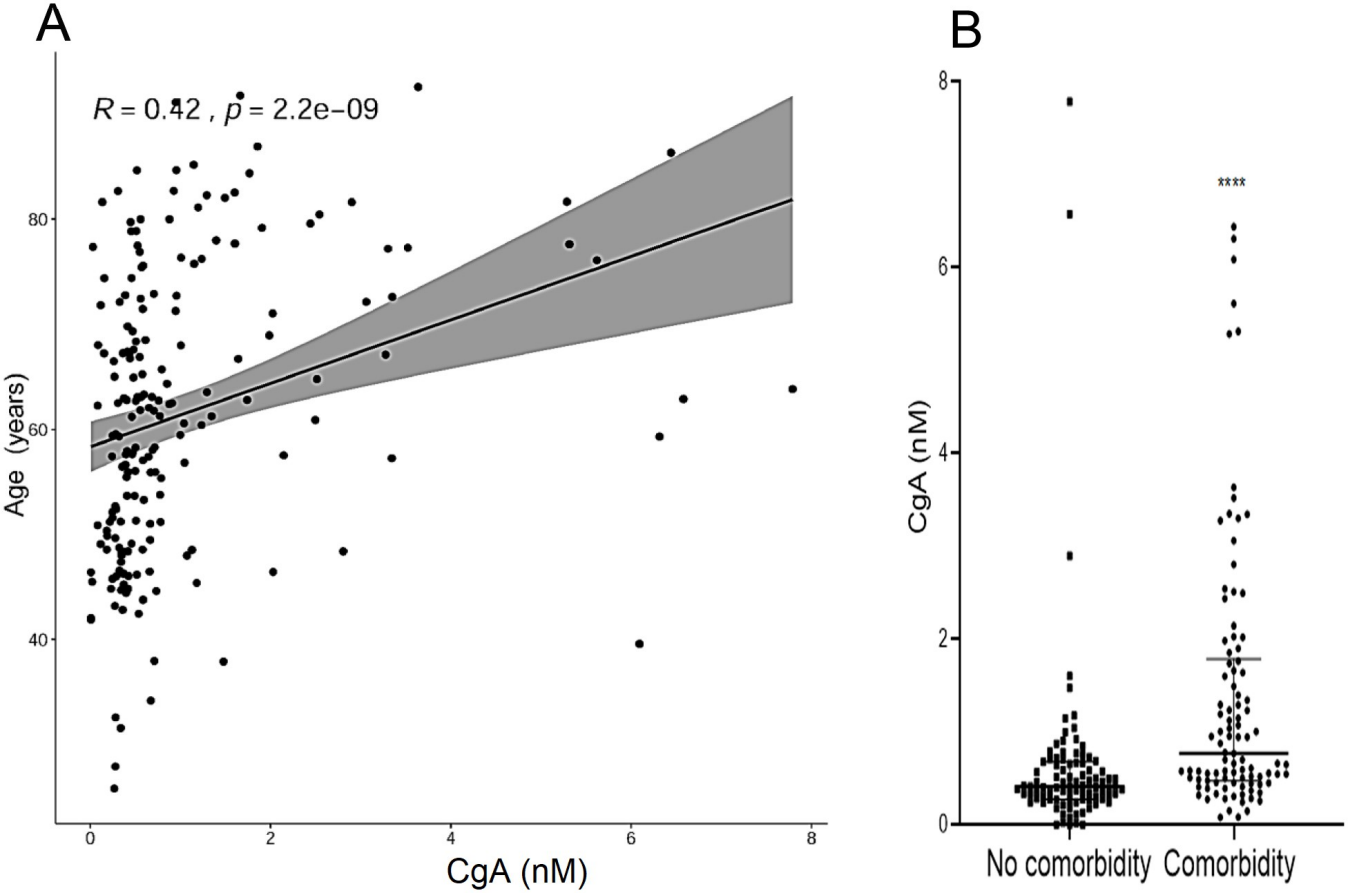
Panel A: Correlation of CgA plasma levels with age. Panel B: CgA plasma levels in patients with or without comorbidities. *** <0.0001.

Plasma levels of CgA were significantly higher in COVID-19 patients compared with HC (0.558 nM [0.358–1.046] vs 0.368 nM [0.288–0.490] respectively, p = 0.0017, Fig 1, panel **A**). Similarly, VS-I plasma levels were more elevated in patients than in HC (0.357 [0.196–0.465] nM vs 0.144 [0.144–0.156] nM respectively, p<0.0001, Fig 1, panel **A**). The CgA and V76 plasma levels were similar in male and female patients (0.5190 [0.351–0.926] nM and 0.6705 [0.36975–1.439] nM for CgA and 0.3505 [0.185–0.45125] nM and 0.3660 [0.2505–0.492] nM for V76). CgA levels at admission correlated with age (R 0.42, p<0.0001, Fig 2, panel **A**) and were higher in patients with at least a single pre-existing comorbidity compared with non-comorbid patients (0.778 [0.483–1.761] nM vs 0.419 [0.281–0.675] nM respectively, p<0.0001, Fig 2, panel **B**). CgA or VS-I plasma levels did not differ between females and males (*not shown*). CgA correlated with the degree of hypoxia, as quantified by PaO_2_/FiO_2_ (R -0.20, p = 0.0057) and with CRP (R 0.30, p<0.0001), NLR (R 0.21. p = 0.0062) and LDH levels (R 0.17, p = 0.017). No significant correlation was observed between VS-I concentration and age, comorbidities, CRP or LDH levels.

### CgA and VS-I levels in survivors and non-survivors

**Fig 3:**
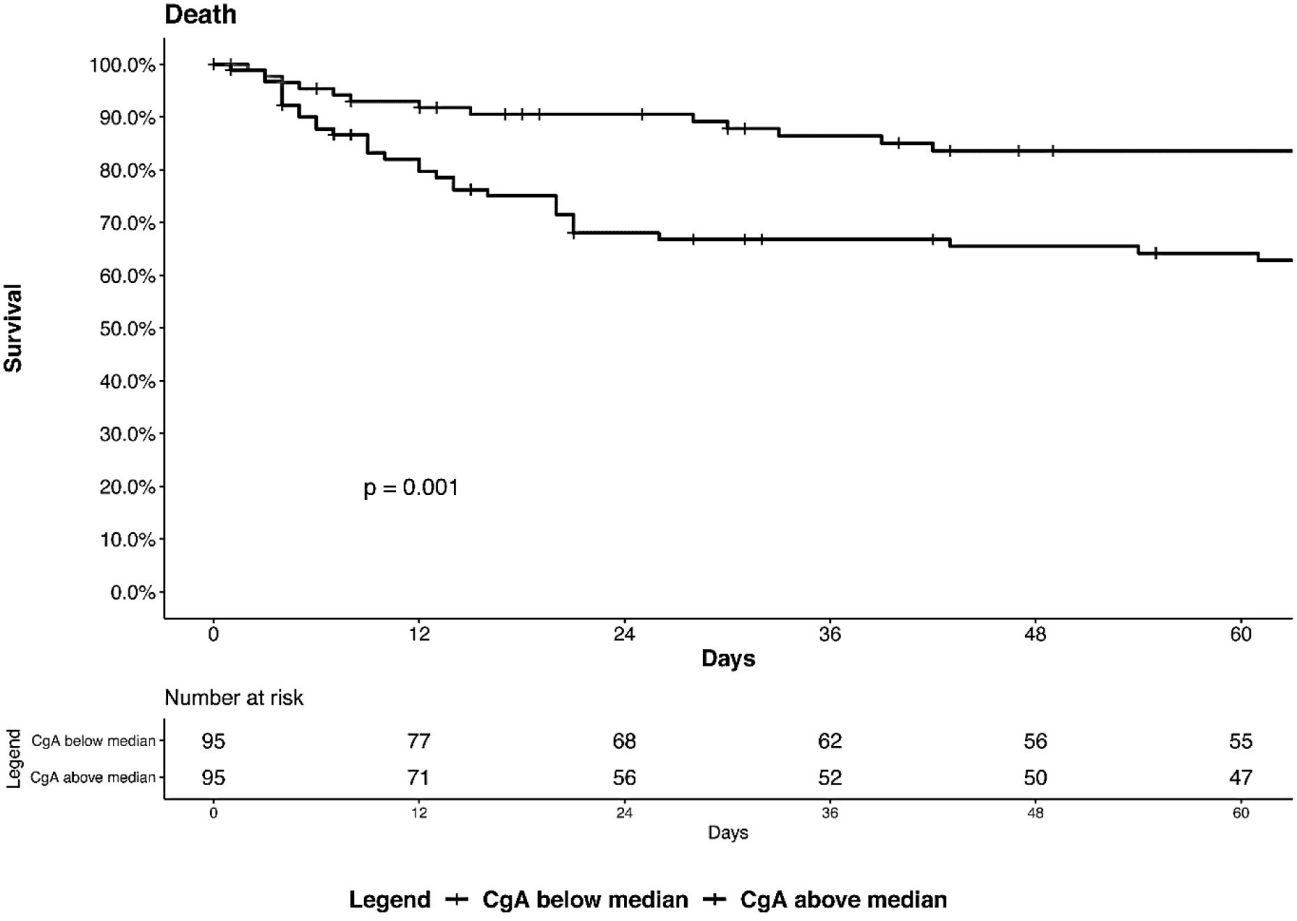
Kaplan-Meier survival curves in patients with CgA levels below (low) or above (high) the median value of 0.558 nM. Log rank test, p = 0.001.

Patients who died had higher plasma levels of CgA at admission than COVID-19 survivors (0.948 [0.514–1.754] nM vs 0.507 [0.343–0.785] nM respectively, p = 0.00026) (Fig 1, panel **B**). In contrast, VS-I plasma levels were similar in survivors and patients who died (p>0.05, Fig 1, panel **B**). At multivariable cox regression analysis, CgA plasma levels at admission were independent predictors of in-hospital mortality when adjusting for age, number of comorbidities, degree of respiratory dysfunction (PaO_2_/FiO_2_), systemic inflammation as reflected by CRP levels at admission and time from symptom onset to sampling (Table 2). Kaplan Meier survival analysis confirmed that patients with levels of CgA above the median value (0.557 nM) at admission had a higher risk of mortality (log rank test, p = 0.001, Fig 3).

**Table 2:** Multivariable Cox regression analysis predicting death.

	HR	95% CI	P value
CgA (nM)	1.23	1.02–1.47	0.025
Age (years)	1.02	0.99–1.05	0.20
Number of comorbidities	1.34	1.01–1.76	0.039
PaO_2_/FiO_2_	0.99	0.99–0.99	0.018
CRP (mg/dL)	1.00	0.99–1.01	0.19
Time from symptom onset to sampling (days)	0.88	0.81–0.95	0.002

Abbreviations: HR, hazard ratio; 95% CI, 95% confidence interval; CgA, chromogranin A; PaO_2_/FiO_2_, ratio of arterial oxygen partial pressure to fractional inspired oxygen; CRP, C-reactive protein

## Discussion

To our knowledge, this is the first report on CgA and VS-1 levels in COVID-19 and the association of this event with clinical outcomes, as evidenced by in-hospital mortality. The host response to SARS-CoV-2 in patients is substantial, with inflammation, vascular activation and coagulopathy playing major roles in the outcome. Neuroendocrine activation is less studied. The observation that CgA accumulates in patients with COVID-19 and that this occurs preferentially in those that eventually die offers a tool to disentangle the immune/neuroendocrine connection in the host response to SARS-CoV-2.

Age and comorbidities are important risk factors for adverse outcome in COVID-19 [26, 32]. We found a correlation between plasma CgA levels and age, and observed that the concentration of CgA was higher in comorbid patients. Nevertheless, CgA was a significant predictor of mortality independently of age and comorbidities. These results are consistent with the association between CgA levels and outcomes in patients with systemic inflammatory response syndrome [13]. Plasma levels of VS-I are increased in critically ill patients [14]. In contrast VS-I plasma levels in COVID-19 patients, although higher than in HC, were similar in survivors and non-survivors. However, CgA and VS-I exert different physiopathological roles, especially in the context of the cardiovascular and immune systems [33]. VS-1 is a product of CgA N-terminal proteolytic processing. Several enzymes are involved, depending on the site of action and on the pathophysiological conditions [5, 6]. We found that in COVID-19 patients the extent of CgA release into the blood predicts clinical outcome. In contrast, the proteolytic processing of CgA yielding VS-I did not change in patients with severe outcome, suggesting that the molecular machinery involved in the generation of VS-I is similarly regulated in COVID-19 patients regardless of disease progression.

CgA levels correlated with hypoxia and with systemic inflammation. Increased values of CgA have been previously described in inflammatory and autoimmune disorders. CgA in turn controls the response to cytokines of the endothelium [34]. CgA influences the vascular remodeling in stress conditions, directly and through the generation of bioactive fragments [35, 36]. In particular, full length CgA induces in endothelial cells the protease nexin-1, an antiangiogenic protein [37, 38]. Nexin-1 is also an inhibitor of plasmin and thrombin [39]. This inhibition might prevent the cleavage of CgA by these enzymes, thereby preserving its activity. SARS-CoV-2 infection and consequent inflammation induce endothelial damage, platelet activation, thrombosis, microangiopathy and neo-angiogenesis in response to tissue injury [40, 41]. Platelet derived microparticles [42] and high levels of angiopoietin-2, follistatin and PAI-1, markers of endothelial injury, increase the risk of mortality [43], and signs of intussusceptive angiogenesis, a proposed mechanism for vessel generation in late stages of chronic lung injury, have been found in lung biopsies of COVID-19 patients [44]. Thus, CgA accumulation in patients with severe COVID-19 might affect the microvascular response to the SARS-CoV-2 infection, possibly influencing the clinical outcome.

We did not identify the origin and mechanisms of CgA production in COVID-19 patients. Potential mechanisms include an enhanced stress-induced sympathetic tone and neuroendocrine activation, leading to enhanced secretion of CgA. Several other mechanisms might contribute to the size of the circulating pool of CgA, including reduced clearance of the molecule itself, viral-induced changes in neurosecretory cells activity and injury, and, at least for those patients under PPI treatment, drug-induced production of CgA by enterochromaffin-like cells or damage of secretory cells [45].

We included 40 age- and sex-matched healthy controls. The relatively small size of samples from healthy controls, which were collected prior to the pandemic outbreak, was forced by the rapid spread of SARS-CoV-2 infection and the systematic vaccination against SARS-CoV-2, events that may represent potential bias for data interpretation.

CgA plasma levels are not gender-dependent [46], but daily fluctuations in CgA levels may occur [47]. Circulating CgA levels may be also altered by treatment with PPI [31, 48]. In our cohort, a minority of patients were on chronic PPI therapy at the time of blood withdrawal. Whether the observed increase in CgA levels in non-survivors is at least in part related to PPI therapy remains uncertain. However, the finding of higher levels of CgA also in patients not receiving PPI compared with HC and the relatively low prevalence of PPI therapy in the cohort imply that additional mechanisms other than PPI therapy are responsible for CgA overexpression. Starting from 2020, several signals have been identified as players in the natural history of the disease. Accordingly, potential biomarkers and predictors of clinical outcome have been proposed (e.g. see [49–54]) with several characteristics of blood cells, inflammatory signals and pathways, biomarkers of innate and acquired immunity, altered cell metabolism and coagulation providing valuable information for dissecting patients heterogeneity [49]. Our results demonstrate for the first time the involvement of CgA, a prototype member of the granin glycoprotein family produced and released by a wide range of different cells throughout the body in the response to SARS-CoV-2 infection. Further studies are needed to identify the role played by CgA and the possibility that the signal can be used for early stratification of patients based on the risk of adverse outcome.

## Conclusions

This study provides the first evidence of an elevation of CgA and its fragment VS-I in COVID-19 patients suggesting a neuroendocrine activation in these patients. CgA levels (but not VS-I) predicts the risk of death independently of other risk factors for adverse outcome. CgA plasma level at hospital admission could therefore represent a tool for the early identification of patients at increased risk of unfavorable disease evolution and therefore needing a more intense management.

## Abbreviations

95% CI: 95% confidence interval
CAD: coronary artery disease
CgA: chromogranin A
COVID-19: Coronavirus disease 2019
CRP: C-reactive protein
DM: diabetes mellitus
FiO_2_: fractional inspired oxygen
HTN: arterial hypertension
ICU: intensive care unit
IQR: interquartile range
LDH: lactate dehydrogenase
NLR: neutrophil to lymphocyte ratio
PaO_2_: arterial oxygen partial pressure
PPI: proton-pump inhibitors
VS-I: vasostatin I
